# Extracellular matrix of dental pulp stem cells: applications in pulp tissue engineering using somatic MSCs

**DOI:** 10.3389/fphys.2013.00395

**Published:** 2014-01-06

**Authors:** Sriram Ravindran, Chun-Chieh Huang, Anne George

**Affiliations:** Brodie Tooth Development Genetics and Regenerative Medicine Research Laboratory, Department of Oral Biology, University of Illinois at ChicagoChicago, IL, USA

**Keywords:** biomimetics, extracellular matrix, 3D scaffold, pulp tissue regeneration, dental pulp stem cells, periodontal ligament stem cells, human marrow stromal cells

## Abstract

Dental Caries affects approximately 90% of the world's population. At present, the clinical treatment for dental caries is root canal therapy. This treatment results in loss of tooth sensitivity and vitality. Tissue engineering can potentially solve this problem by enabling regeneration of a functional pulp tissue. Dental pulp stem cells (DPSCs) have been shown to be an excellent source for pulp regeneration. However, limited availability of these cells hinders its potential for clinical translation. We have investigated the possibility of using somatic mesenchymal stem cells (MSCs) from other sources for dental pulp tissue regeneration using a biomimetic dental pulp extracellular matrix (ECM) incorporated scaffold. Human periodontal ligament stem cells (PDLSCs) and human bone marrow stromal cells (HMSCs) were investigated for their ability to differentiate toward an odontogenic lineage. *In vitro* real-time PCR results coupled with histological and immunohistochemical examination of the explanted tissues confirmed the ability of PDLSCs and HMSCs to form a vascularized pulp-like tissue. These findings indicate that the dental pulp stem derived ECM scaffold stimulated odontogenic differentiation of PDLSCs and HMSCs without the need for exogenous addition of growth and differentiation factors. This study represents a translational perspective toward possible therapeutic application of using a combination of somatic stem cells and extracellular matrix for pulp regeneration.

## Introduction

Dental caries is the most prevalent infectious disease among children and adults. Approximately 90% of the world's population has experienced dental caries (Petersen et al., 2005). Dental caries is characterized by infected and necrotic dental pulp tissue. The dental pulp tissue provides vitality and sensitivity to the tooth. The pulp tissue is highly vascularized, innervated and also serves as a source of stem cells. These characteristics enable the pulp to play a significant role in homeostasis and formation of reparative dentin (Schmalz and Galler, 2011). Current clinical treatment for dental caries is root canal therapy. This involves the cleaning and replacement of the infected and necrotic pulp tissue with a mineral trioxide compound. As a result of replacing a living tissue with a trioxide compound, the tooth looses its vitality and sensitivity and it is prone to secondary infections and the complications associated with it (Cordeiro et al., 2008). In adolescents, root canal treatment poses an even greater problem by preventing root maturation (Lentzari and Kozirakis, 1989; McTigue et al., 2013).

All of these deficiencies can be overcome by the use of tissue engineering strategies to regenerate the dental pulp. The identification of stem cells from several dental tissues has made pulp tissue regeneration a realistic clinical possibility. The identified dental stem cells include: Dental pulp stem cells (DPSCs) (Gronthos et al., 2000), periodontal ligament derived stem cells (PDLSCs) (Gould et al., 1977; Gronthos et al., 2006), stem cells from the root apical papilla (SCAP) (Sonoyama et al., 2006), and stem cells from exfoliated deciduous teeth (SHED) (Miura et al., 2003). All of these mesenchymal stem cells (MSCs) have been shown to be multi-potent and capable of differentiation into different cell types of the mesenchymal lineage.

In order to engineer the dental pulp tissue successfully, the choice of stem cells, scaffolds and growth factors is paramount. Several recent publications, have shown the ability of different dental cell types with both natural and artificial polymeric scaffolds and growth factors to regenerate dental pulp-like tissue in a subcutaneous implantation model (Cordeiro et al., 2008; Alsanea et al., 2011; Huang, 2011; Sakai et al., 2011). However, more recently, CD105 positive and CD31 negative dental pulp cells along with collagen and stromal derived factor 1 (SDF1) were used to regenerate the dental pulp in a canine pulpectomy model (Iohara et al., 2011; Ishizaka et al., 2012). Additionally, Wang et al. showed the preliminary potential of dental pulp cells in regenerating pulp-like tissue in canine immature teeth (Wang et al., 2013).

Although all of these studies demonstrate promise, from a clinical perspective, the prospect of retrieving autologous dental stem cells for multiple pulp regeneration therapies is daunting (Demarco et al., 2011). The DPSCs in adult humans are limited to the availability of the third molars and are not replenished after extraction like the bone marrow. Most clinical cases possess more than one carious tooth. Additionally, the prospect of obtaining a sub population of stem cells is even more difficult. However, if it is possible to use other stem cell sources or multiple mesenchymal cells in combination, dental pulp tissue regeneration can be a clinically viable technique. The major drawback to this ideology is that MSCs from different sources require a different set of growth factors to achieve lineage specific differentiation. Additionally, the safety, amount and timing of delivery of the growth factors pose a significant challenge. As a first step toward this goal, it is necessary to ascertain that MSCs from different sources can undergo odontogenic differentiation under similar conditions.

We have shown that it is possible to generate biomimetic ECM incorporated scaffolds containing the intact ECM of any cell type. The ECM is unique for each cell type and governs tissue architecture, growth factor delivery and cellular behavior *in vivo*. Our published data indicates that the ECM embedded scaffold can be used to achieve lineage specific differentiation of MSCs *in vitro* and *in vivo* without the need for exogenous growth factor delivery (Ravindran et al., 2010, 2013). This strategy is clinically relevant as the biomimetic scaffolds can be generated using human cell lines and hence can be produced in large quantities. Autologous somatic stem cells from the patient can then be used to achieve tissue specific regeneration making this approach patient specific without complications of immune response. In this study, we explore the possibility of achieving odontogenic differentiation of PDLSCs and HMSCs for dental pulp tissue regeneration. These findings can be an important part of a clinically translatable platform for therapeutic regeneration of pulp tissue.

## Materials and methods

### Cell culture

Three different cell types were used in this study namely, HMSCs, DPSCs, and PDLSCs. HMSCs were obtained from the Tulane Cancer Center. The human DPSCs and PDLSCs were a gift from Dr. Songtao Shi (University of Southern California). All the three cell types were cultured in minimum essential medium alpha (αMEM), (GIBCO) containing 20% fetal bovine serum (FBS), (GIBCO), 1% L-glutamine, (GIBCO) and 1% antibiotic and antimycotic solution, (GIBCO). This will be referred to as growth media throughout this manuscript.

### Generation of ECM scaffolds

The dental pulp stem cell derived ECM scaffolds were generated as per published protocols (Ravindran et al., 2013). Briefly, DPSCs were cultured within collagen/chitosan scaffolds under the influence of differentiation media The prepared scaffolds were stored in Hank's balanced salt solution (HBSS) containing 5% antibiotic and antimycotic solution. The scaffolds were used within a week of being generated. For DPP blocking, the scaffolds were incubated overnight at 4°C in 500 μ l of DPP antibody (1/100) or rabbit IgG (1/100). They were washed 4× in HBSS prior to cell seeding.

### Live-dead cell assay

DPSCs, PDLSCs, and HMSCs (2 × 10^6^ per scaffold) were cultured within the ECM scaffolds for up to 2 weeks in standard cell culture conditions in the presence of growth media. At 24 h, 1 week, and 2 weeks post seeding, live and dead cells in triplicate samples were analyzed using the live-dead cell assay kit (Molecular Probes) as per the manufacturer's recommended protocol. The calcein AM and the ethidium homodimer concentrations were 10 μ M each to indicate the live (green) and dead (red) cells respectively. The samples were imaged using a Zeiss Axioobserver D1 fluorescent microscope equipped with the required filter sets. Five individual fields of view were imaged for each and the percentage of dead cells to live cells was calculated.

### Confocal microscopy

DPSCs, PDLSCs, and HMSCs were seeded within the ECM scaffolds as described previously. Twenty-four hours, 1 week, and 2 weeks post seeding, triplicate samples were fixed in 4% formalin, permeablized with 0.5% triton-x100 for 1 h and stained with phalloidin probe conjugated with TRITC fluorescence probe to visualize the actin fibers. The samples were then z-stack imaged using a Zeiss LSM 710 confocal microscope. 3D renderings were produced using the Zeiss Zen imaging software.

### Scanning electron microscopy (SEM)

DPSC, PDLSCs, and HMSCs were seeded within the ECM scaffolds as described above. They were fixed in 4% formalin, dehydrated in graded ethanol solutions, dried, coated with gold/palladium and imaged using a Hitachi SU8030 SEM.

### Quantitative real time PCR

HMSCs were cultured within the ECM scaffolds or control collagen/chitosan scaffolds in quadruplicate for 2 weeks using growth media. After 2 weeks, RNA was isolated using the Qiagen RNA isolation kit as per the manufacturer's instructions. First strand synthesis was performed using superscript III and the generated cDNA was subjected to gene specific quantitative PCR amplification. Fold change was calculated using the comparative delta Ct method. Statistical significance between the control and ECM samples were calculated using student's *t*-test. A greater that 95% confidence interval was deemed significant. Table 1 lists the gene specific primer sequences used in this study.

**Table 1:** **Gene specific primers**.

**Gene**	**Primer sequence**	
	**Forward 5′–3′**	**Reverse 5′–3′**
Runx2	TGGTTACTGTCATGGCGGGTA	TCTCAGATCGTTGAACCTTGCTA
Osteocalcin	CACTCCTCGCCCTATTGGC	CCCTCCTGCTTGGACACAAAG
VEGFA	AGGGCAGAATCATCACGAAGT	AGGGTCTCGATTGGATGGCA
FGF1	ACACCGACGGGCTTTTATACG	CCCATTCTTCTTGAGGCCAAC
FGF2	AGAAGAGCGACCCTCACATCA	CGGTTAGCACACACTCCTTTG
Coll1A1	GAGGGCCAAGACGAAGACATC	CAGATCACGTCATCGCACAAC
GDF10	AGATCGTTCGTCCATCCAACC	GGGAGTTCATCTTATCGGGAACA
DSPP	TTTGGGCAGTAGCATGGGC	CCATCTTGGGTATTCTCTTGCCT
TGFβ1	CAATTCCTGGCGATACCTCAG	GCACAACTCCGGTGACATCAA
TGFβ2	CAGCACACTCGATATGGACCA	CCTCGGGCTCAGGATAGTCT
MMP2	GATACCCCTTTGACGGTAAGGA	CCTTCTCCCAAGGTCCATAGC
BMP2	ACTACCAGAAACGAGTGGGAA	GCATCTGTTCTCGGAAAACCT
GAPDH	ACAACTTTGGTATCGTGGAAGG	GCCATCACGCCACAGTTTC
PHEX	GAGGCACTCGAATTGCCCT	ACTCCTGTTTAGCTTGGAGACTT

*This table lists the forward and reverse gene specific primer sequences used for qPCR*.

### Subcutaneous implantation

DPSCs, PDLSCs, and HMSCs were seeded at a density of 2 × 10^6^ cells/scaffold in triplicates within control collagen/chitosan scaffold, the ECM scaffold, the DPP blocked ECM scaffold (DPSCs only) and the IgG blocked ECM scaffolds as described previously. The DPP blocked scaffolds were prepared by incubating the odontogenic ECM scaffolds with a DPP antibody as published previously (Ravindran et al., 2013). The DPP antibody is an in-house made antibody. The antibody concentration was 2 mg/ml and it was used at a dilution of 1/100. Rabbit IgG blocked scaffolds served as controls. The cell seeded scaffolds were cultured *in vitro* for 48 h and were then implanted subcutaneously on the back of athymic nude mice (Charles River Laboratories). Four weeks post implantation, the animals were sacrificed and the scaffolds were retrieved, fixed in 4% neutral buffered formalin, embedded and sectioned into 5 μm thick sections for histological evaluation. All animal experiments were performed as per protocol approved by the UIC animal care committee (Assurance number A-3460-01).

### Histology and immunohistochemistry

Hematoxylin and Eosin (H&E) staining was performed as per published protocols (Ravindran et al., 2010, 2013). Alizarin red staining to visualize calcium deposition was performed as per standard procedures. Immunohistochemistry using peroxidase conjugated secondary antibodies and fluorescent probes was performed as per previously published protocols (Ravindran et al., 2010, 2012). The following antibodies were used: Mouse anti-DMP1 antibody, (1/2000, a gift from Dr. Chunlin Qin from Baylor College of Dentistry), rabbit anti-DSP antibody, (1/250, in house generated antibody), rabbit anti-DPP antibody (1/100, in house generated antibody), rabbit anti-VEGF antibody (1/100, Santa Cruz Biotechnology), mouse anti-von Willebrand factor (VWF) antibody (1/100, Santa Cruz Biotechnology). All fluorescently stained sections were imaged using a Zeiss LSM 710 confocal microscope and peroxidase stained sections were imaged using a Zeiss axioobserver D1 microscope. All comparative fluorescence images using the confocal microscope were imaged using the same imaging conditions.

## Results

### Adhesion and survival of mesenchymal cells on biomimetic scaffold

**Figure 1:**
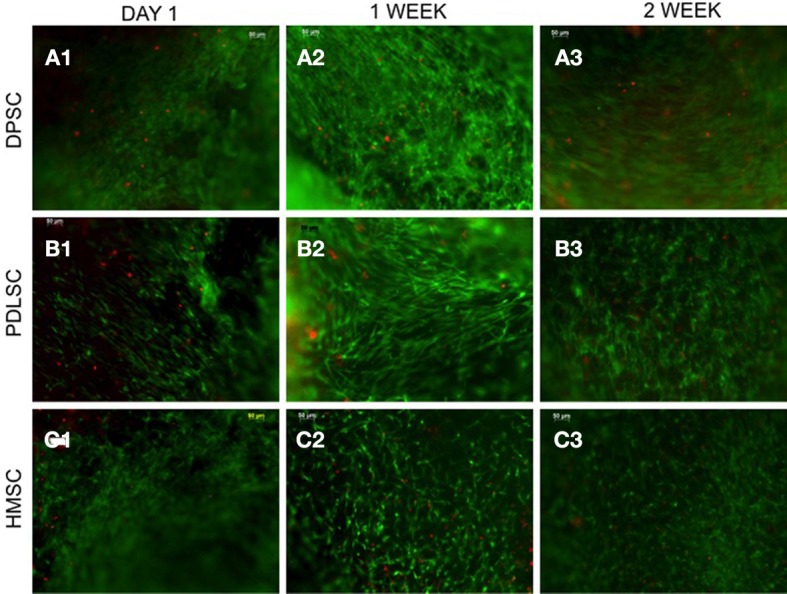
**Live-dead cell assay**. The fluorescent micrographs are representative images of DPSCs **(A)**, PDLSCs **(B)**, and HMSCs **(C)** within the pulp ECM scaffolds imaged after 24 h **(A1–C1)**, 1 week **(A2–C2)**, and 2 weeks **(A3–C3)** post seeding. The green fluorescent cells represent live cells and the red fluorescent cells represent dead cells. All the images were imaged under the same magnification and the scale bar in all the images represents 50 μm.

A principle requirement for a scaffold in tissue engineering is that it must promote cell adhesion, survival, and interaction with the ECM. The cell-matrix adhesion and survival of DPSCs, PDLSCs, and HMSCs within the pulp ECM scaffolds were evaluated. Figure 1 shows representative images from the live-dead cell assay for up to 2 weeks post seeding. Green staining in these images represents live cells and red staining represents dead cells. It is evident from the figures that all three cell types survived over a period of 2 weeks. Quantitative analysis of the fluorescent images indicated that the percentage of live cells in the three cell types studied at 24 h, 1 week, and 2 weeks exceeded 98%. Additionally, there was no statistically significant difference between the percentages of live cells between time points for the same cell type or between different cell types at the same time point.

**Figure 2:**
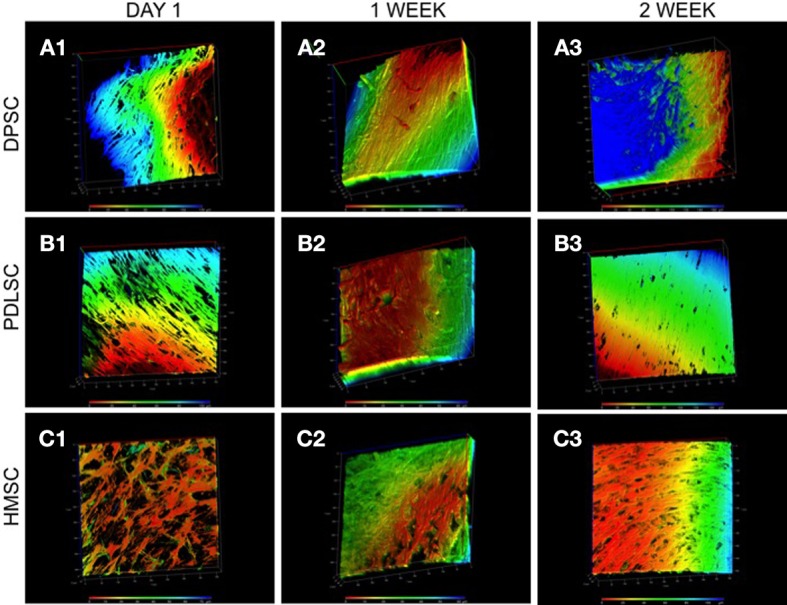
**3D confocal microscopy of DPSCs, PDLSCs, and HMSCs in the pulp ECM scaffolds**. The fluorescent images are reconstructed 3D images of a z-stack of confocal images that represent the arrangement of DPSCs **(A)**, PDLSCs **(B)**, and HMSCs **(C)** after 24 h **(A1–C1)**, 1 week **(A2–C2)**, and 2 weeks **(A3–C3)** post seeding. The images are color coded for depth indicating the 3 dimensional orientations of the cells. The scale at the bottom represents the scale for color-coding based on depth (μ m). The boxes surrounding the image represent the x, y, and the z-axes and are scaled in μ m.

Fluorescent actin staining followed by 3D confocal imaging was performed to evaluate the three dimensional orientation of the three cell types in the pulp ECM scaffold. The images in Figure 2 are color coded for depth. It is evident from the figure that DPSCs, PDLSCs, and HMSCs show a three dimensional orientation with cell processes spanning several microns in all three dimensions. Figure 2 also provides a qualitative overview of the proliferation of the three cell types in the pulp ECM scaffold. An increase in cell density can be observed between A1 and A3, B1 and B3, and C1 and C3.

**Figure 3:**
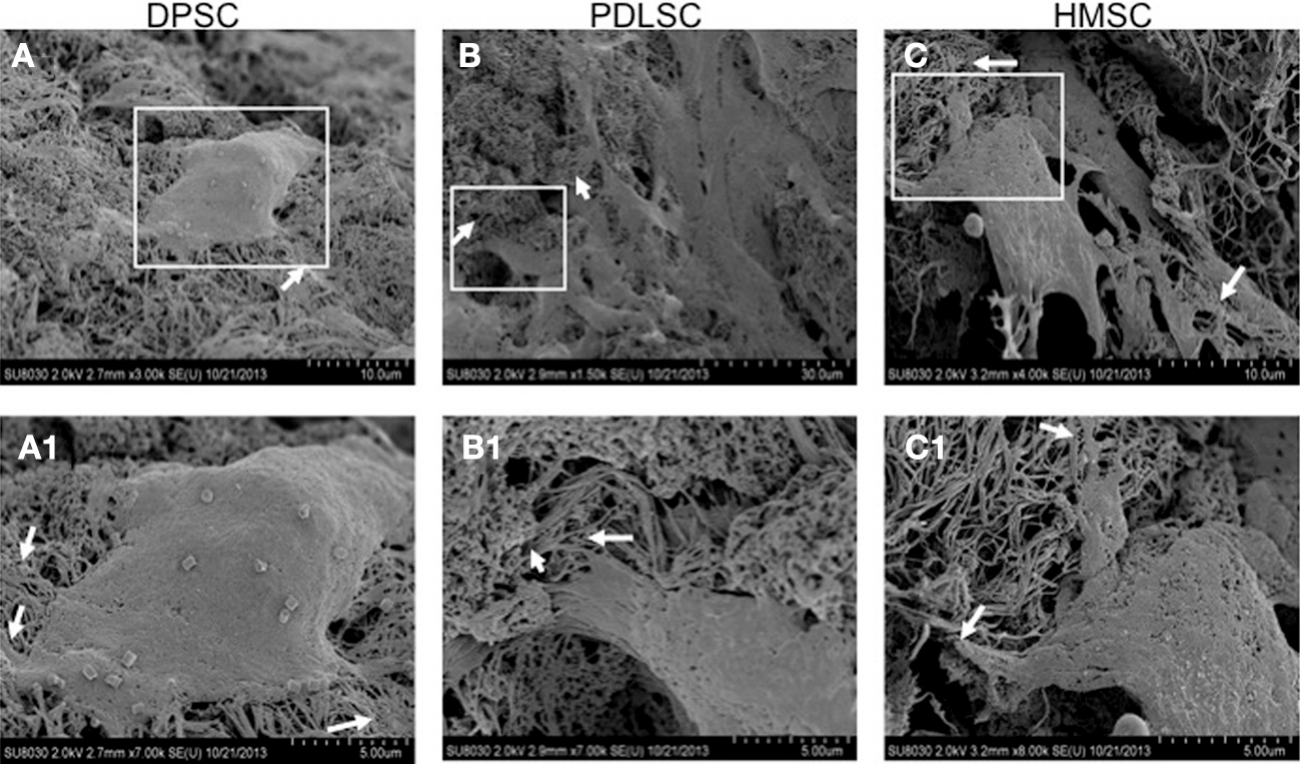
**Scanning electron microscopy**. The SE micrographs are representative images of DPSCs **(A)**, PDLSCs **(B)**, and HMSCs **(C)** in the pulp ECM scaffolds. The boxed areas in **(A–C)** are represented in higher magnification in **(A1–C1)** respectively. The arrows in all the images point to areas where cellular processes branch out to interact with the matrix.

SEM imaging was performed to study the ultrastructural characteristics of the cells grown within the pulp ECM scaffolds. Results in Figure 3 show pronounced cell-matrix interactions of the three cell types with pulp ECM scaffold. The arrows in Figure 3 point to cell processes interacting with the scaffold.

Collectively, these experiments demonstrated that the DPSCs, PDLSCs, and HMSCs adhered, survived, proliferated, and interacted with their extracellular environment when cultured within the pulp ECM scaffolds.

### *In vitro* odontogenic differentiation of HMSCs within the pulp ECM scaffold

**Figure 4:**
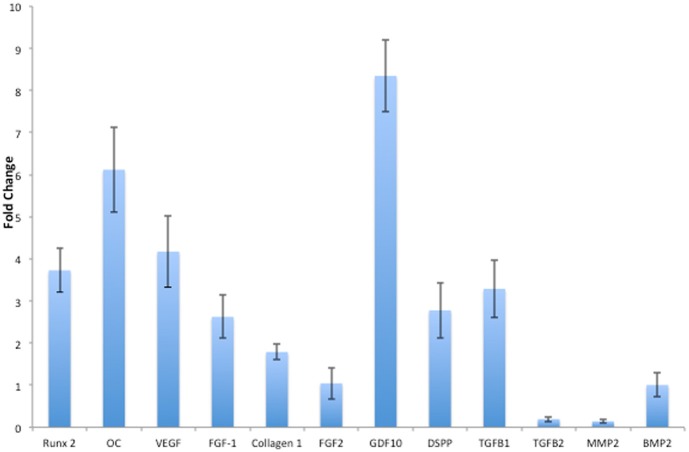
**Quantitative real time PCR analysis**. The bars represent mean fold change in gene expression by the HMSCs cultured within the pulp ECM scaffolds with respect to HMSCs cultured within control collagen/chitosan scaffolds. The experiments were performed in quadruplicates. The error bars represent standard deviation. The statistical significance was calculated using student's *t*-test and the *p*-value is represented individually. Note that FGF2 and BMP2 expression did not show any statistically significant change.

Real time Quantitative PCR was used to analyze the expression of several differentiation marker genes that can be a predictor of odontogenic differentiation of HMSCs. Figure 4 is a graphical representation of the fold change in the expression profile of several marker genes along with their statistical significance. Notably, when HMSCs were cultured within the pulp ECM scaffold, they showed significantly higher expression of odontogenic differentiation markers such as Runx2, osteocalcin (OC), and type I collagen. Importantly, DSPP, a key marker of odontogenic differentiation was up-regulated ~3-fold. The average *Ct* values for DSPP expressed by HMSCs when cultured in the control and pulp ECM scaffolds were 32.83 and 30.97 respectively (average GAPDH values were around 25). This suggests that although there was a significant increase in DSPP expression, the gene expression level was low. A longer time point showed increased DSPP gene expression in PDLSCs (Ravindran et al., 2013). Therefore, HMSCs might require a longer time frame for showing increased expression levels of DSPP gene.

Growth factors TGFβ 1, FGF-1, VEGF, and GDF10 were highly expressed. TGFβ 2 was negatively regulated and BMP2 and FGF2 did not show a statistically significant change in expression. Additionally, phosphate regulating X-lined neutral endopeptidase (PHEX) that was not expressed by the HMSCs cultured within the control scaffolds (*Ct* value undetermined in 40 cycles) was expressed with an average *Ct* value of 32.66 (*n* = 3) by the HMSCs cultured within the pulp ECM scaffold indicating that this phosphate regulating gene expression was turned on.

### Comparison of gene expression data between DPSCs, PDLSCs, and HMSCs after 2 weeks of culture within the ECM scaffolds

Table 2 shows the expression levels of a few genes that were significantly regulated at the 2-week time point by at least 2 cell types. The data presented for DPSCs and PDLSCs are published data (Ravindran et al., 2013). Comparing the expression levels, it can be noted that the three stem cell types regulate their differentiation via different mechanisms. PHEX gene did not show a statistically significant regulation after 2 weeks in DPSCs and PDLSCs. However, the basal expression level of this gene was higher in DPSCs and PDLSCs cultured within the control scaffolds with an average *Ct* value of 31.83 and 28.01 respectively (*n* = 3).

**Table 2:** **List of differentially expressed genes**.

**Gene**	**Fold change**		
	**DPSC**	**PDLSC**	**HMSC**
RUNX2	1.53	1.87	3.73
VEGF	5.46	2.05	4.17
COLL1A1	5.45	No change	1.79
TGFβ1	1.57	2.15	3.29
TGFβ2	−1.92	1.41	−5.0
MMP2	4.34	2.89	−7.14
BMP2	3.2	10.19	No change

*This table lists the expression levels of the specified genes by DPSCs, PDLSCs, and HMSCs after 2 weeks of culture within the ECM scaffolds in the presence of growth media. The DPSC and PDLSC gene expression data are from our published results (Ravindran et al., 2013)*.

### The pulp ECM scaffold can induce expression of odontogenic marker proteins in DPSCs and PDLSCs *In vivo*

**Figure 5:**
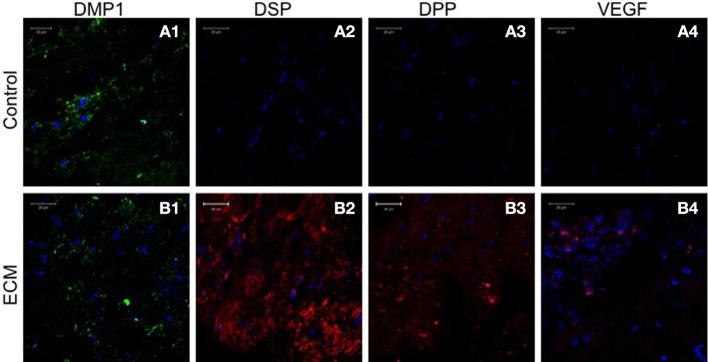
**DPSC immunohistochemistry**. The fluorescent images are representative confocal micrographs of sections from DPSC seeded scaffold explants consisting of control collagen/chitosan scaffold **(A)** and pulp ECM scaffold **(B)**. The sections were analyzed for the expression of DMP1 **(A1,B1)**, DSP **(A2,B2)**, DPP **(A3,B3)**, and VEGF **(A4,B4)**. Note the absence of DSP, DPP, and VEGF signal in **(A)** and its presence in **(B)**. The imaging conditions were maintained constant. The scale bar represents 20 μm in all images.

**Figure 6:**
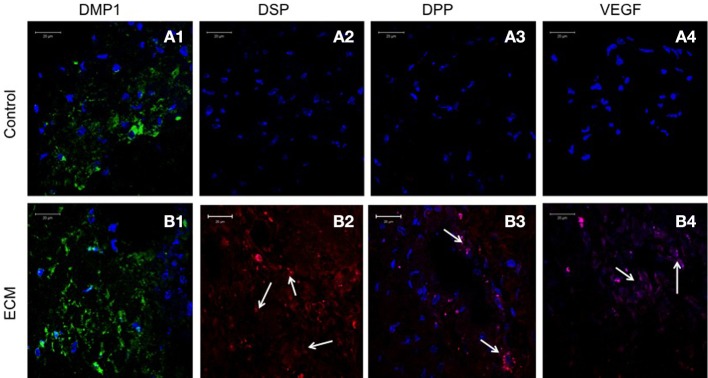
**PDLSC immunohistochemistry**. The fluorescent images are representative confocal micrographs of sections from PDLSC seeded scaffold explants consisting of control collagen/chitosan scaffold **(A)** and pulp ECM scaffold **(B)**. The sections were analyzed for the expression of DMP1 **(A1,B1)**, DSP **(A2,B2)**, DPP **(A3,B3)**, and VEGF **(A4,B4)**. Note the absence of DSP, DPP, and VEGF signal in **(A)** (control scaffolds containing PDLSCs). Arrows in **(B)** point to positive staining of the same by PDLSCs seeded within the pulp ECM scaffold. The imaging conditions were maintained constant. The scale bar represents 20 μm in all images.

**Figure 7:**
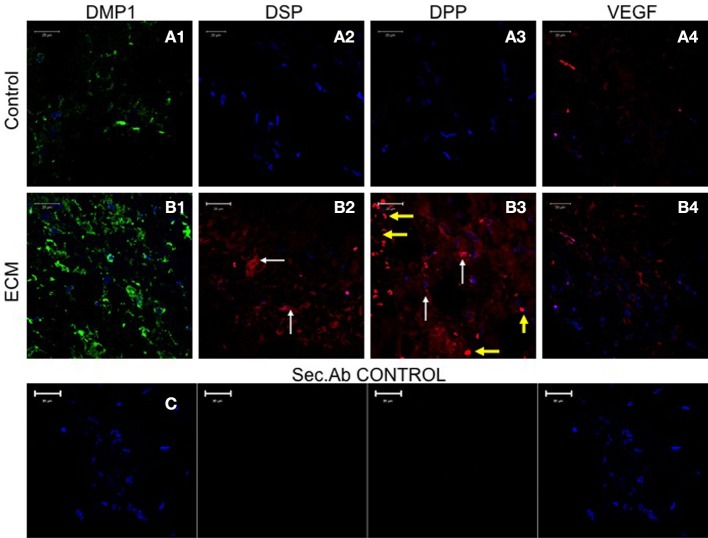
**HMSC immunohistochemistry**. The fluorescent images are representative confocal micrographs of sections from HMSC seeded scaffold explants consisting of control collagen/chitosan scaffold **(A)** and pulp ECM scaffold **(B)**. The sections were analyzed for the expression of DMP1 **(A1,B1)**, DSP **(A2,B2)**, DPP **(A3,B3)**, and VEGF **(A4,B4)**. Note the absence of DSP and DPP signal in **(A)** (control scaffolds containing HMSCs). White arrows in **(B)** point to positive staining of the same by HMSCs seeded within the pulp ECM scaffold. Yellow arrows in **(B3)** point to non-specific fluorescence from red blood corpuscles. Note the increase in DMP1 (comparing images **A1** and **B1**) and VEGF (comparing images **A4** and **B4**) expression. The secondary antibody control for rabbit and mouse secondary antibodies did not show any staining **(C)**. The imaging conditions were maintained constant. The scale bar represents 20 μm in all images.

Sections from pulp ECM scaffolds and control scaffolds containing PDLSCs and DPSCs after 4 weeks of implantation were analyzed by immunohistochemistry for odontogenic marker proteins DMP1, DSP, and DPP. The sections were also analyzed for the expression of VEGF, the pro-angiogenic growth factor. Images in Figures 5, 6 show that in the control scaffold sections, the PDLSCs and DPSCs do not show immunohistochemically detectable levels of DSP, DPP, and VEGF expression (Figures 5, 6A2–A4). However, expression of all the three proteins was observed in the sections from the pulp ECM scaffolds containing PDLSCs and DPSCs (Figures 5, 6B2–B4 with arrows pointing to positive staining). No significant change in DMP1 protein expression levels could be observed in DPSCs (comparing Figures 5A1,B1). A small increase in DMP1 protein expression was observed in PDLSCs cultured within the ECM scaffold (Comparing Figures 6A1,B1). No non-specific fluorescence was observed in rabbit and mouse secondary antibody controls (Figure 7C).

### The pulp ECM scaffold can induce expression of odontogenic marker proteins in HMSCs *In vivo*

Sections from control and pulp ECM scaffolds containing HMSCs were analyzed by immunohistochemistry for the expression of DMP1, DSP, DPP, and VEGF. Figure 7 shows the result of this experiment (performed in triplicates). Comparing images in Figures 7A,B, it is evident that HMSCs expressed DSP and DPP proteins when implanted with the pulp ECM scaffolds (Figures 7B2,B3) and not the control scaffolds (Figures 7A2,A3). The white arrows in the images show the expression of these proteins. The yellow arrows in Figure 7B3 indicate non-specific fluorescence from the red blood corpuscles. Incidentally, this image also serves to show that the pulp ECM scaffolds also promoted vascularization *in vivo*. Additionally, an increase in the protein expression levels of DMP1 and VEGF was also noted (Comparing Figures 7A1–A4 with Figures 7B1–B4). No non-specific fluorescence was observed in rabbit and mouse secondary antibody controls (Figure 7C).

### Calcium deposition within the scaffolds

**Figure 8:**
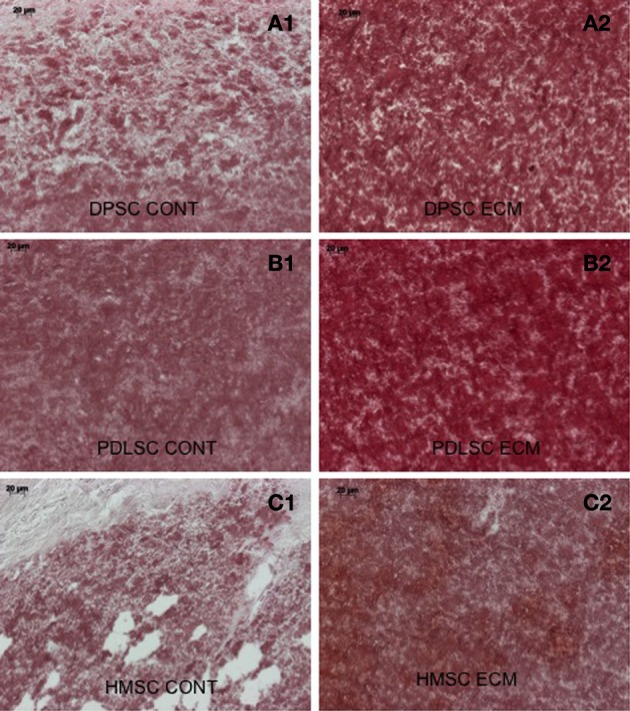
**Alizarin red staining**. The micrographs are representative images of explant sections containing DPSCs **(A)**, PDLSCs **(B)**, and HMSCs **(C)** in control collagen/chitosan scaffolds **(A1–C1)** and pulp ECM scaffolds **(A2–C2)**. Note the increased calcium deposition when the three cell types are cultured within the pulp ECM scaffolds (comparing **A1**, **B1**, and **C1** with **A2**, **B2**, and **C2** respectively). The scale bar in all the images represents 20 μm.

Calcium deposition within the control and ECM scaffolds implant sections was analyzed qualitatively using alizarin red staining. Figure 8 shows sections of the pulp ECM scaffold sections containing DPSCs, PDLSCs, and HMSCs. A stronger alizarin red staining in the ECM scaffolds containing all the three cell types showed increased amount of calcium deposition (Figures 8A2,B2,C2) when compared with the respective control sections (Figures 8A1,B1,C1) under the same imaging conditions. Additionally, in the DPP blocked pulp ECM sections containing DPSCs, the calcium deposition was increased with respect to the control sections (comparing Figures 8A1,A3). However, the expression was reduced with respect to the untreated pulp ECM scaffold sections containing DPSCs (comparing Figures 8A2,A3).

### The pulp ECM scaffolds induced vascularization with DPSCs, PDLSCs, and HMSCs *In vivo*

**Figure 9:**
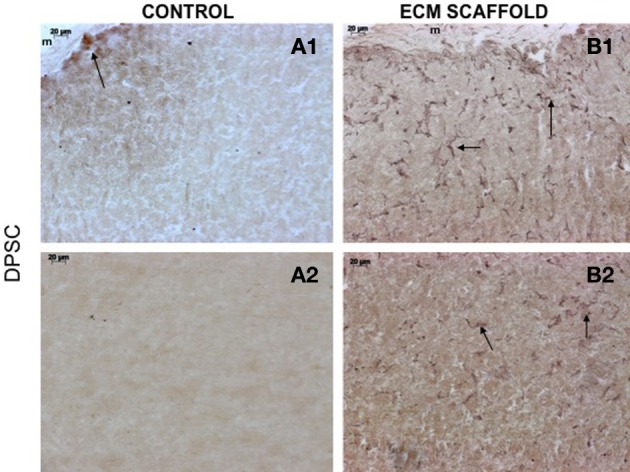
**DPSCs in ECM scaffold: von Willebrand factor (VWF) immunohistochemistry**. The micrographs are representative images of VWF stained explant sections containing DPSCs in control **(A)** and pulp ECM **(B)** scaffolds. In all the images, 1 and 2 nomenclature indicates micrographs that show expression of VWF in the periphery (1) and within (2) the respective scaffolds. The arrows in the images point to positively stained endothelial cells and capillary networks. Note the absence of positive staining in the interior of the control scaffolds containing DPSCs **(A2)**, and positive staining in the periphery of the control scaffolds **(A1)**.

**Figure 10:**
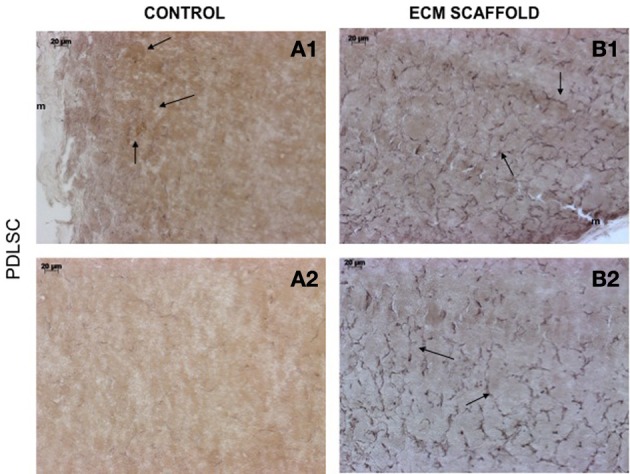
**PDLSCs in ECM scaffold: von Willebrand factor (VWF) immunohistochemistry**. The micrographs are representative images of VWF stained explant sections containing PDLSCs **(A,B)** in control and pulp ECM scaffolds. In all the images, 1 and 2 nomenclature indicates micrographs that show expression of VWF in the periphery (1) and within (2) the respective scaffolds. The arrows in the images point to positively stained endothelial cells and capillary networks. Note the absence of positive staining in the interior of the control scaffolds containing PDLSCs **(A2)**, and positive staining in the periphery of the control scaffolds **(A1)**. The scale bar in all the images represents 20 μm.

**Figure 11:**
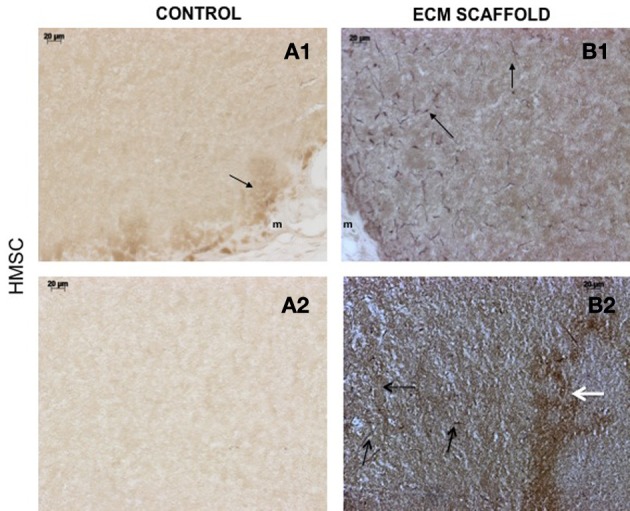
**HMSCs in ECM scaffold: von Willebrand factor (VWF) immunohistochemistry**. The micrographs are representative images of VWF stained explant sections containing HMSCs **(A,B)** in control and pulp ECM scaffolds. In all the images, 1 and 2 nomenclature indicates micrographs that show expression of VWF in the periphery (1) and within (2) the respective scaffolds. The arrows in the images point to positively stained endothelial cells (black arrows) and capillary networks (white arrows). Note the absence of positive staining in the interior of the control scaffolds containing HMSCs **(A2)**, and positive staining in the periphery of the control scaffolds **(A1)**. Also note the presence of capillary networks in the pulp ECM scaffolds. The scale bar in all the images represents 20 μm.

von Willebrand factor (VWF), a marker for endothelial cells was used to look for the presence of endothelial cells and thereby, capillary networks in the explant sections of the control and pulp ECM scaffolds containing DPSCs, PDLSCs, and HMSCs. Figures 9–11 shows representative images of the results from this experiment. Overall, we observed that in the control scaffolds (plain collagen/chitosan scaffolds) that do not contain embedded ECM, endothelial cells were present in the periphery of the scaffolds for all the three cell types [Figure 9A1 (DPSCs), 10A1 (PDLSCs), and 11A1 (HMSCs)] showing peripheral migration of host endothelial cells. However, endothelial cells were not observed within these scaffolds for all the three cell types (Figures 9A2, 10A2, 11A2).

On the other hand, in the pulp ECM scaffolds containing all the three cell types, more endothelial cells were present in the periphery (Figures 9B1, 10B1, 11B1). Additionally, endothelial cells were also observed within the scaffolds (Figures 9B2, 10B2, 11B2, black arrows). In the explant sections of ECM scaffolds containing HMSCs, in addition to individual endothelial cells (black arrows in Figure 11B2), we were also able observe the presence of capillary-like networks positively stained by VWF antibody within the scaffold. These networks showed a different staining pattern when compared to individual endothelial cells and are indicated by a white arrow in Figure 11B2. Together, these results suggest that the pulp ECM scaffolds can trigger vascularization irrespective of the mesenchymal cell type present within.

## Discussion

DPSCs are the ideal stem cell source to achieve dental pulp tissue regeneration. Despite the importance of these cells, their limited availability hinders their use in regenerative applications. In our earlier study, we had shown that it is possible to regenerate pulp-like tissue expressing odontogenic markers DSP, DPP, and DMP1 using DPSCs and biomimetic pulp ECM scaffolds *in vitro* and *in vivo* (Ravindran et al., 2013). In this study we have verified that the biomimetic ECM scaffold maintains lineage specific differentiation of DPSCs over a period of 4 weeks and we have identified the odontogenic differentiation potential of other somatic stem cells such as PDLSCs and HMSCs.

As a significant step toward realizing the potential of the ECM mediated pulp tissue engineering as clinical possibility, we have explored the use of PDLSCs and HMSCs for dental pulp tissue regeneration. The results from our study indicate that the three stem cells used in this study: DPSC, PDLSCs, and HMSCs showed excellent viability and proliferated to populate the scaffold when seeded within the biomimetic ECM scaffolds. SEM images indicated the presence of cell-matrix interactions confirming the ability of the biomimetic pulp ECM scaffolds to support the attachment and proliferation of multiple MSCs. This information is important from the perspective of developing the biomimetic scaffolds as a tool that can support multiple MSCs in combination at the same time.

Extracellular matrix (ECM) provides cells with cues for differentiation. We have previously shown that the PDLSCs can undergo odontogenic differentiation *in vitro* when cultured within the biomimetic ECM scaffold. In the present study, we show that PDLSCs can undergo and maintain odontogenic differentiation *in vivo* over a period of 4 weeks. Data from immunohistochemical analysis with respect to DMP1, DSP, and DPP expression and alizarin red staining for the presence of calcium indicated that the pulp ECM scaffolds could induce the PDLSCs to differentiate toward an odontogenic lineage. More importantly, we show that HMSCs can undergo odontogenic differentiation without stimulus from external growth factors both *in vitro* and *in vivo*. The quantitative gene expression data indicated that HMSCs, when cultured within the pulp ECM scaffold, showed statistically significant upegulation of growth factors, transcription factors and signaling molecules involved in differentiation. Runx2, a key transcription factor involved in odontogenesis (James et al., 2006) was significantly upregulated. Additionally, pro-osteogenic/odontogenic growth factors involved in craniofacial development such as FGF1 (Nie et al., 2006), TGFβ 1(Nie et al., 2006), and GDF10(Nie et al., 2006) were significantly upregulated. As a result of these events, an increase in the expression of type I collagen that is the major constituent of the pulp and the dentin matrix was also observed. Significant upregulation of DSPP transcript followed by protein expression as seen in the immunohistochemistry data indicated that the HMSCs were differentiating toward an odontogenic lineage. On the other hand, we have observed that HMSCs cultured on osteogenic ECM scaffolds do not show DSPP expression (Ravindran et al., 2012), lending more proof to our hypothesis that tissue specific ECM can direct and maintain lineage specific differentiation of stem cells.

Upon evaluation of our published data (Ravindran et al., 2013) and the data presented in this manuscript, it is possible to observe the differential behavior of the three cell types toward a common goal. For example, growth factors such as BMP2 and TGFβ 1 and metalloproteases like MMP2 were differentially regulated by the three MSCs. Extrapolating from our data, we can conclude that although these three different MSCs possess the same potential for differentiation into multiple lineages, they react differently in response to extracellular stimuli. Ultimately, all three stem cell types studied, showed expression of marker proteins DSP and DPP. However, their mode of regulation was markedly different. In DPSCs, DSPP gene expression did not show any statistically significant difference after 2 weeks of *in vitro* culture in the ECM scaffold. However, there was a change in protein expression as observed by immunohistochemistry (Ravindran et al., 2013). On the other hand, PDLSCs showed increased DSPP gene expression after 4 weeks of culture within the ECM scaffold (Ravindran et al., 2013) and the HMSCs showed increased gene expression after 2 weeks of culture within the ECM scaffolds. However, all three cell types showed protein expression of DSP and DPP *in vivo*. This disparity suggests that the expression of this key marker protein is regulated both transcriptionally and translationally in the three stem cell types studied and not essentially in the same manner.

Another important observation in this study was that the HMSCs, when cultured within the ECM scaffolds, were able to “turn on” the expression of PHEX, a phosphate regulating protein that is important for the differentiation of DPSCs. Mutations in PHEX can cause X-linked familial hypophospahatemic rickets (Rowe et al., 2005). A recent study has also shown that abnormal PHEX can lead to impaired DPSC differentiation (Salmon et al., 2013). Therefore, it was encouraging to see the expression of PHEX by HMSCs. This fact, coupled with the histological evaluation results indicates that the HMSCs can undergo odontogenic differentiation *in vitro* and *in vivo*. On the other hand, the expression of the PHEX gene was not regulated with statistical significance in DPSCs and PDLSCs at the 2-week time point. In the DPSCs and PDLSCs, PHEX was expressed with an average *Ct* value of 31.83 and 28.01 in the control scaffolds. However, in the HMSC controls, PHEX was not expressed at all. Considering these differences, it is an important observation that PHEX was turned on in the HMSCs when cultured within the odontogenic ECM scaffolds. We hypothesize that since PHEX was already being expressed in the other two cell types, its gene expression profile remained unchanged at this time point when cultured within the odontogenic ECM scaffolds.

Angiogenesis is vital for pulp tissue regeneration. We have shown that the pulp ECM scaffolds are enriched with VEGF (Ravindran et al., 2013). Results from our experiments show that the pulp ECM scaffolds can trigger vascularization irrespective of the stem cell type seeded within the scaffolds. The presence of capillary networks evidenced by VWF staining and the upregulation of VEGF, a pro-angiogenic growth factor serve as proof. This is an important result from a translational perspective that indicates that it is possible to use other MSC sources for pulp tissue regeneration without affecting the vascularization potential of the scaffolds.

Overall, this study shows that the biomimetic pulp ECM scaffolds can induce odontogenic differentiation of three different MSCs namely: DPSCs, PDLSCs, and HMSCs both *in vitro* and *in vivo*. This study also proves that it is possible to achieve ECM mediated lineage specific differentiation of multiple somatic MSCs without the need for complex growth factor delivery systems. In conclusion, the pulp ECM embedded biomimetic scaffolds can provide a clinically translatable platform for dental pulp tissue repair and regeneration. Future studies will focus on pulp tissue regeneration using these scaffolds in the pulp chamber and also target the possibility of using multiple stem cells within the pulp ECM scaffold to evaluate the ability of the biomimetic scaffold to induce collective lineage specific differentiation.

## Author contributions

Sriram Ravindran: First author. Conceptualized, planned, performed all the experiments and wrote the manuscript. Chun-Chieh Huang: Second author. Planned animal model experiments with Sriram Ravindran, performed animal surgery and subsequent analysis involving histology and immunohistochemistry with Sriram Ravindran. Anne George: Corresponding author. Was involved in conceptualization and planning with Sriram Ravindran. Contributed toward editing and proofreading the manuscript along with Sriram Ravindran.

### Conflict of interest statement

The authors declare that the research was conducted in the absence of any commercial or financial relationships that could be construed as a potential conflict of interest.
